# Aqua­bis­[2,5-bis­(pyridin-2-yl)-1,3,4-thia­diazole-κ^2^ *N* ^2^,*N* ^3^](trifluoro­methane­sulfonato-κ*O*)copper(II) trifluoro­methane­sulfonate

**DOI:** 10.1107/S1600536812008732

**Published:** 2012-03-03

**Authors:** Fouad Bentiss, Moha Outirite, Michel Lagrenée, Mohamed Saadi, Lahcen El Ammari

**Affiliations:** a Laboratoire de Chimie de Coordination et d’Analytique (LCCA), Faculté des Sciences, Université Chouaib Doukkali, BP 20, M-24000 El Jadida, Morocco; b Unité de Catalyse et de Chimie du Solide (UCCS), CNRS UMR 8181, ENSCL, BP 90108, F-59652 Villeneuve d’Ascq Cedex, France; c Université Lille Nord de France, F-59000 Lille, France; d Laboratoire de Chimie du Solide Appliquée, Faculté des Sciences, Université Mohammed V-Agdal, Avenue Ibn Battouta, BP. 1014, Rabat, Morocco

## Abstract

2,5-Bis(pyridin-2-yl)-1,3,4-thia­diazole (denoted *L*) has been found to act as a bidentate ligand in the monomeric title complex, [Cu(CF_3_O_3_S)(C_12_H_8_N_4_S)_2_(H_2_O)](CF_3_O_3_S). The complex shows a distorted octahedrally coordinated copper(II) cation which is linked to two thia­diazole ligands, one water mol­ecule and one trifluoro­methane­sulfonate anion. The second trifluoro­methane­sulfonate anion does not coordinate the copper(II) cation. Each thia­diazole ligand uses one pyridyl and one thia­diazole N atom for the coordination of copper. The N atom of the second non-coordinating pyridyl substituent is found on the same side of the 1,3,4-thia­diazole ring as the S atom. The trifluoro­methane­sulfonate ions are involved in a three-dimensional network of O—H⋯O hydrogen bonds. C—H⋯N inter­actions also occur.

## Related literature

For the synthesis of the ligand, see: Lebrini *et al.* (2005 ▶). For background to compounds with the same ligand but other metals and other counter-anions, see: Bentiss *et al.* (2002 ▶, 2004 ▶, 2011*a* ▶,*b* ▶); Keij *et al.* (1984 ▶); Zheng *et al.* (2006 ▶).
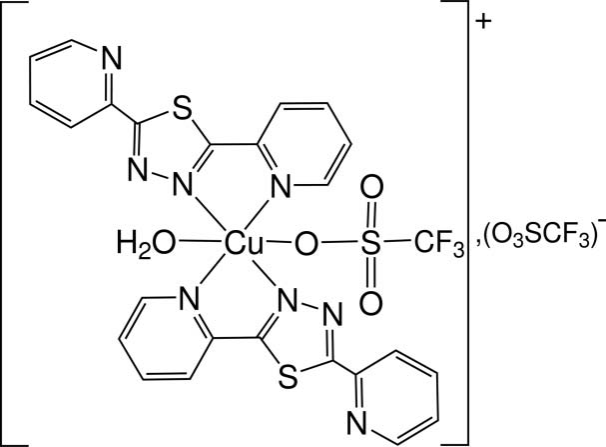


## Experimental

### 

#### Crystal data

[Cu(CF_3_O_3_S)(C_12_H_8_N_4_S)_2_(H_2_O)](CF_3_O_3_S)
*M* *_r_* = 860.26
Triclinic, 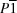

*a* = 8.469 (3) Å
*b* = 11.116 (3) Å
*c* = 18.834 (6) Å
α = 92.111 (14)°
β = 90.823 (14)°
γ = 107.352 (14)°
*V* = 1690.6 (9) Å^3^
*Z* = 2
Mo *K*α radiation
μ = 0.98 mm^−1^
*T* = 100 K
0.39 × 0.30 × 0.17 mm

#### Data collection

Bruker APEXII CCD diffractometer
Absorption correction: multi-scan (*SADABS*; Sheldrick, 1995 ▶) *T*_min_ = 0.879, *T*_max_ = 1.000
12380 measured reflections
6517 independent reflections
5421 reflections with *I* > 2σ(*I*)
*R*_int_ = 0.031

#### Refinement

*R*[*F* ^2^ > 2σ(*F* ^2^)] = 0.041
*wR*(*F* ^2^) = 0.099
*S* = 1.03
6517 reflections
469 parameters
H-atom parameters constrained
Δρ_max_ = 1.39 e Å^−3^
Δρ_min_ = −0.41 e Å^−3^

### 

Data collection: *APEX2* (Bruker, 2005 ▶); cell refinement: *SAINT* (Bruker, 2005 ▶); data reduction: *SAINT*; program(s) used to solve structure: *SHELXS97* (Sheldrick, 2008 ▶); program(s) used to refine structure: *SHELXL97* (Sheldrick, 2008 ▶); molecular graphics: *ORTEP-3 for Windows* (Farrugia, 1997 ▶) and *ORTEPIII* (Burnett & Johnson, 1996 ▶); software used to prepare material for publication: *WinGX* (Farrugia, 1999 ▶).

## Supplementary Material

Crystal structure: contains datablock(s) I, global. DOI: 10.1107/S1600536812008732/im2358sup1.cif

Structure factors: contains datablock(s) I. DOI: 10.1107/S1600536812008732/im2358Isup2.hkl

Additional supplementary materials:  crystallographic information; 3D view; checkCIF report

## Figures and Tables

**Table 1:** Hydrogen-bond geometry (Å, °)

*D*—H⋯*A*	*D*—H	H⋯*A*	*D*⋯*A*	*D*—H⋯*A*
O1—H1*W*⋯O6	0.78	1.95	2.721 (3)	169
O1—H2*W*⋯O2^i^	0.80	1.94	2.732 (3)	167
C6—H6⋯N7	0.95	2.33	3.146 (4)	143
C18—H18⋯N3	0.95	2.36	3.174 (4)	143

Symmetry code: (i) 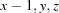.

**Fig. 1.**
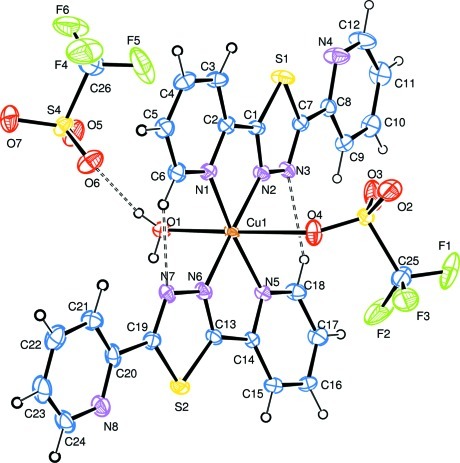
Molecular structure of the title compound showing displacement ellipsoids at the 50% probability level. H atoms are represented as small circles. Hydrogen bonds are depicted as dashed lines.

**Fig. 2.**
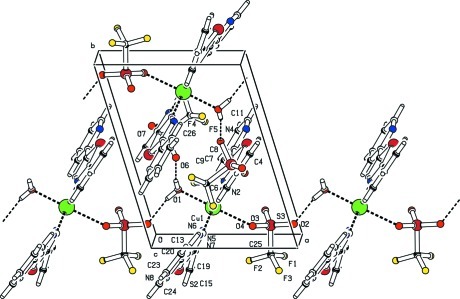
Plot of the unit cell showing a packing diagram. Hydrogen bonds are depicted as dashed lines.

## supplementary crystallographic information

### Comment

With ligands containing five-membered nitrogen heterocycles, 3 d transition metals such as Ni(II) and Cu(II) have a tendency to form mono- or polynuclear species (Keij *et al.*, 1984). Dinuclear species are of interest due to the potential magnetic coupling of unpaired 3 d electrons *via* bridging nitrogen containing ligands. Ligands related to 1,2-diazoles with *o*-pyridine substitution at position 3 and 5, such as 2,5-bis(2-pyridyl)-1,3,4-oxadiazole and thiadiazole, have been of interest for such applications. Indeed, 2,5-bis(2-pyridyl)-1,3,4-thiadiazole can be used in transition metal complexes in association with additional anionic ligands. In the resulting di- and mononuclear complexes, a variety of coordination modes have been observed, of which the dinuclear (N`N``, N2, N``) bridging, the dinuclear (N`N``, N2, N``)_2_ double bridging and the mononuclear (N`,*N*`)_2_ coordination mode are the most common and most important ones (Scheme 1). The latter mode in octahedral complexes is exclusively observed in *trans* configuration. For the dimeric mode, we have previously reported the synthesis and characterization of the corresponding complexes of Cu(II) and Ni(II) with the 2,5-bis(2-pyridyl)-thiadiazole derivative (bptd) (Bentiss *et al.*, 2004). There are no other reports of the dimeric structures of solid state complexes of this neutral ligand (bptd).

The structures of monomeric complexes of the neutral 2,5-bis(2-pyridyl)-1,3,4-thiadiazole derivative with divalent Zn (chloride and perchlorate), Co (nitrate, perchlorate and tetrafluoborate), Ni (perchlorate and tetrafluoborate), and Cu (nitrate, perchlorate) have been previously reported (Bentiss *et al.*, 2002; Bentiss *et al.*, 2011*a*; Zheng *et al.*, 2006; Bentiss *et al.*, 2011*b*). We report here the synthesis and the single-crystal structure of the new monomeric complex formed by 2,5-bis(2-pyridyl)-1,3,4-thiadiazole with copper trifluoromethanesulfonate.

In the new monomeric title complex, the Cu atom is no longer situated on a center of symmetry: its octahedral coordination sphere is built from two crystallographically independent molecules *L* and two O atoms of different chemical entities: O1 is from a water molecule with Cu1—O1 = 2.259 (2) Å and O4 from one trifluoromethanesulfonate anion with a very long distance Cu1—O4 = 2.540 (3)Å (Fig.1). The axial distortion of the octahedron corresponds to the Jahn-Teller effect typical for Cu^2+^. While N—Cu—O1 angles range from 88.18 (9)° (N2—Cu—O1) to 94.74 (9)° (N1—Cu—O1), keeping O1 at the axial position on one side of the distorted equatorial plane, the bonded O4 trifluoromethanesulfonate end is located in the opposite axial position, with N—Cu—O4 angles ranging from 85.90 (9)° (N5—Cu—O4) to 89.47 (9)° (N6—Cu—O4).

In this monomeric complex, a completely different ligand configuration is observed compared to our recently reported Co and Ni monomeric complexes of bptd. In both *L* ligands the non-complexed pyridyl rings are still coplanar with the central thiadiazole heterocycle, while both complexed pyridyl rings are no longer coplanar with the central thiadiazole. In one of the ligands *L*, topped with the CF_3_ end of the Cu bound trifluoromethanesulfonate, a small interplanar angle of 3.7 (2) ° of the pyridyl moiety with the thiadiazole ring is observed. On the other hand, in the second ligand this twist is much more pronounced as indicated by an interplanar angle of 12.8 (2)° of the non-coordinated pyridine witrh respect to the remaining planar part of *L.* This difference cannot be related to any hydrogen bonding interaction with its neighbouring unbound trifluoromethanesulfonate. The trifluoromethanesulfonate ions are involved in an infinite three-dimensional network of O–H···O hydrogen bonds (see Fig.2).

### Experimental

2,5-Bis(2-pyridyl)-1,3,4-thiadiazole ligand (noted *L*) was synthesized as described previously (Lebrini *et al.*, 2005). Cu(O_3_SCF_3_)_2_ (1.5 mmol, 0.54 g) in 8 ml of water was added to (0.42 mmol, 0.1 g) of *L* (bptd ligand) dissolved in 8 ml of ethanol. The solution was filtered and after 24 h, the blue compound crystallized at room temperature. Yield: 63%. Crystals were washed with water and dried under vacuum. Anal. Calc. for C_25_H_18_CuF_6_N_8_O_7_S_4_: C, 36.27; H, 2.09; N, 13.02; S, 14.91; F, 13.25%. Found: C, 36.32; H, 2.17; N, 12.98; S, 14.88; F, 13.30%.

### Refinement

H atoms were located in a difference map and treated as riding with C—H = 0.95 Å for the aromatic CH, with *U*_iso_(H) = 1.2 *U*_eq_(C). The O-bound H atoms were initially also located in a difference map and refined with O—H distance restraints of 0.86 (1). In a the last cycle they were refined using the riding model approximation with *U*_iso_(H) set to 1.2U_eq_(O).

### Crystal data

**Table:**

[Cu(CF_3_O_3_S)(C_12_H_8_N_4_S)_2_(H_2_O)](CF_3_O_3_S)	*Z* = 2
*M**_r_* = 860.26	*F*(000) = 866
Triclinic, *P*1	*D*_x_ = 1.690 Mg m^−^^3^
Hall symbol: -P 1	Mo *K*α radiation, λ = 0.71073 Å
*a* = 8.469 (3) Å	Cell parameters from 4698 reflections
*b* = 11.116 (3) Å	θ = 2.5–28.2°
*c* = 18.834 (6) Å	µ = 0.98 mm^−^^1^
α = 92.111 (14)°	*T* = 100 K
β = 90.823 (14)°	Irregular parallelepiped, blue
γ = 107.352 (14)°	0.39 × 0.30 × 0.17 mm
*V* = 1690.6 (9) Å^3^	

### Data collection

**Table:**

Bruker APEXII CCD diffractometer	6517 independent reflections
Radiation source: fine-focus sealed tube	5421 reflections with *I* > 2σ(*I*)
Graphite monochromator	*R*_int_ = 0.031
φ and ω scans	θ_max_ = 26.0°, θ_min_ = 2.7°
Absorption correction: multi-scan (*SADABS*; Sheldrick, 1995)	*h* = −10→10
*T*_min_ = 0.879, *T*_max_ = 1.000	*k* = −12→13
12380 measured reflections	*l* = −19→23

### Refinement

**Table:**

Refinement on *F*^2^	Primary atom site location: structure-invariant direct methods
Least-squares matrix: full	Secondary atom site location: difference Fourier map
*R*[*F*^2^ > 2σ(*F*^2^)] = 0.041	Hydrogen site location: difference Fourier map
*wR*(*F*^2^) = 0.099	H-atom parameters constrained
*S* = 1.03	*w* = 1/[σ^2^(*F*_o_^2^) + (0.0306*P*)^2^ + 2.8618*P*] where *P* = (*F*_o_^2^ + 2*F*_c_^2^)/3
6517 reflections	(Δ/σ)_max_ = 0.001
469 parameters	Δρ_max_ = 1.39 e Å^−^^3^
0 restraints	Δρ_min_ = −0.41 e Å^−^^3^

### Special details

**Table:**

Geometry. All s.u.'s (except the s.u. in the dihedral angle between two l.s. planes) are estimated using the full covariance matrix. The cell s.u.'s are taken into account individually in the estimation of s.u.'s in distances, angles and torsion angles; correlations between s.u.'s in cell parameters are only used when they are defined by crystal symmetry. An approximate (isotropic) treatment of cell s.u.'s is used for estimating s.u.'s involving l.s. planes.
Refinement. Refinement of *F*^2^ against all reflections. The weighted *R*-factor *wR* and goodness of fit *S* are based on *F*^2^, conventional *R*-factors *R* are based on *F*, with *F* set to zero for negative *F*^2^. The threshold expression of *F*^2^ > 2σ(*F*^2^) is used only for calculating *R*-factors(gt) *etc*. and is not relevant to the choice of reflections for refinement. *R*-factors based on *F*^2^ are statistically about twice as large as those based on *F*, and *R*- factors based on all data will be even larger.

### Fractional atomic coordinates and isotropic or equivalent isotropic displacement parameters (Å^2^)

**Table:**

	*x*	*y*	*z*	*U*_iso_*/*U*_eq_	
C1	0.7108 (3)	0.3893 (3)	0.21546 (15)	0.0167 (6)	
C2	0.7265 (4)	0.3860 (3)	0.29290 (15)	0.0171 (6)	
C3	0.8424 (4)	0.4766 (3)	0.33445 (16)	0.0215 (6)	
H3	0.9241	0.5425	0.3132	0.026*	
C4	0.8363 (4)	0.4688 (3)	0.40768 (16)	0.0244 (7)	
H4	0.9141	0.5290	0.4378	0.029*	
C5	0.7151 (4)	0.3720 (3)	0.43558 (16)	0.0227 (7)	
H5	0.7062	0.3657	0.4856	0.027*	
C6	0.6052 (4)	0.2830 (3)	0.39019 (15)	0.0214 (6)	
H6	0.5235	0.2158	0.4104	0.026*	
C7	0.6958 (4)	0.4195 (3)	0.09165 (15)	0.0177 (6)	
C8	0.7178 (4)	0.4640 (3)	0.01825 (15)	0.0187 (6)	
C9	0.6216 (4)	0.3978 (3)	−0.03813 (16)	0.0230 (7)	
H9	0.5360	0.3219	−0.0309	0.028*	
C10	0.6513 (4)	0.4437 (3)	−0.10520 (17)	0.0275 (7)	
H10	0.5875	0.3995	−0.1452	0.033*	
C11	0.7754 (4)	0.5548 (3)	−0.11309 (16)	0.0293 (7)	
H11	0.7987	0.5887	−0.1587	0.035*	
C12	0.8654 (5)	0.6161 (3)	−0.05375 (18)	0.0358 (9)	
H12	0.9500	0.6931	−0.0600	0.043*	
C13	0.2339 (3)	−0.0673 (3)	0.27519 (15)	0.0175 (6)	
C14	0.2426 (3)	−0.0758 (3)	0.19841 (15)	0.0169 (6)	
C15	0.1621 (4)	−0.1827 (3)	0.15729 (16)	0.0213 (6)	
H15	0.1006	−0.2579	0.1786	0.026*	
C16	0.1736 (4)	−0.1772 (3)	0.08417 (16)	0.0221 (6)	
H16	0.1190	−0.2484	0.0541	0.026*	
C17	0.2662 (4)	−0.0659 (3)	0.05565 (16)	0.0243 (7)	
H17	0.2740	−0.0594	0.0056	0.029*	
C18	0.3478 (4)	0.0364 (3)	0.10069 (16)	0.0225 (6)	
H18	0.4131	0.1116	0.0805	0.027*	
C19	0.1962 (4)	−0.0710 (3)	0.39831 (15)	0.0192 (6)	
C20	0.1420 (4)	−0.0996 (3)	0.47119 (15)	0.0211 (6)	
C21	0.2257 (4)	−0.0288 (3)	0.52982 (17)	0.0256 (7)	
H21	0.3220	0.0405	0.5245	0.031*	
C22	0.1648 (5)	−0.0620 (3)	0.59622 (17)	0.0303 (8)	
H22	0.2179	−0.0160	0.6379	0.036*	
C23	0.0248 (5)	−0.1638 (3)	0.60030 (17)	0.0324 (8)	
H23	−0.0199	−0.1893	0.6452	0.039*	
C24	−0.0505 (4)	−0.2287 (3)	0.53868 (18)	0.0295 (8)	
H24	−0.1477	−0.2978	0.5428	0.035*	
C25	0.7497 (4)	−0.0999 (3)	0.1475 (2)	0.0326 (8)	
C26	0.5085 (5)	0.6738 (3)	0.3395 (2)	0.0406 (9)	
Cu1	0.46280 (4)	0.16646 (3)	0.245633 (17)	0.01429 (10)	
F1	0.8341 (3)	−0.1118 (2)	0.09071 (11)	0.0517 (6)	
F2	0.5891 (3)	−0.1337 (2)	0.12690 (18)	0.0797 (10)	
F3	0.7741 (3)	−0.18338 (19)	0.19345 (14)	0.0505 (6)	
F4	0.5915 (3)	0.6734 (2)	0.40113 (16)	0.0634 (8)	
F5	0.5912 (3)	0.6359 (3)	0.28793 (16)	0.0692 (8)	
F6	0.5078 (3)	0.7939 (2)	0.32673 (17)	0.0646 (8)	
N1	0.6097 (3)	0.2881 (2)	0.31953 (12)	0.0167 (5)	
N2	0.5919 (3)	0.3016 (2)	0.18095 (12)	0.0169 (5)	
N3	0.5828 (3)	0.3191 (2)	0.10989 (12)	0.0170 (5)	
N4	0.8406 (4)	0.5733 (3)	0.01268 (14)	0.0305 (7)	
N5	0.3375 (3)	0.0327 (2)	0.17125 (13)	0.0175 (5)	
N6	0.3392 (3)	0.0282 (2)	0.31048 (12)	0.0183 (5)	
N7	0.3180 (3)	0.0260 (2)	0.38209 (12)	0.0183 (5)	
N8	0.0056 (3)	−0.1997 (3)	0.47406 (14)	0.0253 (6)	
O1	0.2619 (2)	0.26218 (19)	0.24674 (11)	0.0229 (5)	
H1W	0.2659	0.3116	0.2778	0.027*	
H2W	0.1708	0.2137	0.2402	0.027*	
O2	0.9771 (3)	0.0724 (2)	0.21448 (15)	0.0409 (7)	
O3	0.8043 (3)	0.1395 (2)	0.12360 (13)	0.0413 (7)	
O4	0.6920 (3)	0.0630 (2)	0.23593 (11)	0.0261 (5)	
O5	0.2264 (3)	0.5841 (2)	0.27020 (11)	0.0337 (6)	
O6	0.3212 (3)	0.4449 (2)	0.35242 (12)	0.0348 (6)	
O7	0.2270 (3)	0.6206 (2)	0.39735 (11)	0.0320 (6)	
S1	0.82436 (10)	0.50206 (7)	0.16166 (4)	0.02375 (18)	
S2	0.09600 (9)	−0.16694 (7)	0.32694 (4)	0.02022 (17)	
S3	0.81271 (9)	0.06319 (7)	0.18366 (4)	0.01879 (16)	
S4	0.29722 (10)	0.56968 (7)	0.33972 (4)	0.02205 (17)	

### Atomic displacement parameters (Å^2^)

**Table:**

	*U*^11^	*U*^22^	*U*^33^	*U*^12^	*U*^13^	*U*^23^
C1	0.0168 (14)	0.0142 (14)	0.0171 (14)	0.0016 (11)	0.0018 (11)	0.0011 (11)
C2	0.0179 (15)	0.0169 (14)	0.0171 (14)	0.0061 (12)	−0.0003 (11)	0.0011 (11)
C3	0.0207 (15)	0.0187 (15)	0.0212 (15)	0.0002 (12)	−0.0043 (12)	−0.0002 (12)
C4	0.0261 (17)	0.0230 (16)	0.0214 (16)	0.0041 (13)	−0.0088 (13)	−0.0055 (13)
C5	0.0234 (16)	0.0270 (16)	0.0164 (14)	0.0061 (13)	−0.0014 (12)	−0.0029 (12)
C6	0.0224 (16)	0.0238 (16)	0.0165 (14)	0.0048 (13)	0.0015 (12)	−0.0017 (12)
C7	0.0184 (15)	0.0182 (14)	0.0157 (14)	0.0044 (12)	−0.0003 (11)	−0.0016 (11)
C8	0.0232 (16)	0.0176 (14)	0.0168 (14)	0.0077 (12)	0.0061 (12)	0.0047 (11)
C9	0.0223 (16)	0.0213 (15)	0.0242 (16)	0.0052 (13)	0.0001 (13)	−0.0029 (12)
C10	0.0335 (19)	0.0321 (18)	0.0189 (15)	0.0133 (15)	−0.0022 (13)	−0.0009 (13)
C11	0.040 (2)	0.0361 (19)	0.0143 (15)	0.0137 (16)	0.0014 (14)	0.0096 (13)
C12	0.041 (2)	0.0284 (18)	0.0277 (18)	−0.0062 (16)	0.0045 (15)	0.0128 (15)
C13	0.0165 (14)	0.0157 (14)	0.0198 (15)	0.0033 (12)	0.0051 (12)	0.0044 (11)
C14	0.0137 (14)	0.0175 (14)	0.0190 (14)	0.0039 (11)	0.0025 (11)	−0.0010 (11)
C15	0.0187 (15)	0.0179 (15)	0.0243 (16)	0.0007 (12)	0.0020 (12)	−0.0002 (12)
C16	0.0199 (15)	0.0189 (15)	0.0230 (15)	0.0002 (12)	−0.0032 (12)	−0.0069 (12)
C17	0.0243 (16)	0.0276 (17)	0.0180 (15)	0.0037 (13)	−0.0024 (12)	−0.0014 (13)
C18	0.0228 (16)	0.0224 (16)	0.0187 (15)	0.0012 (13)	−0.0012 (12)	0.0034 (12)
C19	0.0218 (15)	0.0170 (14)	0.0192 (15)	0.0060 (12)	0.0035 (12)	0.0019 (11)
C20	0.0263 (16)	0.0243 (16)	0.0176 (15)	0.0142 (13)	0.0090 (12)	0.0075 (12)
C21	0.0258 (17)	0.0215 (16)	0.0285 (17)	0.0049 (13)	0.0047 (14)	0.0044 (13)
C22	0.044 (2)	0.0307 (18)	0.0189 (16)	0.0158 (16)	0.0018 (15)	−0.0017 (13)
C23	0.046 (2)	0.0355 (19)	0.0222 (17)	0.0198 (17)	0.0181 (15)	0.0139 (15)
C24	0.0293 (18)	0.0267 (17)	0.0341 (19)	0.0086 (15)	0.0164 (15)	0.0119 (14)
C25	0.0199 (17)	0.0327 (19)	0.045 (2)	0.0097 (14)	−0.0067 (15)	−0.0156 (16)
C26	0.030 (2)	0.030 (2)	0.065 (3)	0.0115 (16)	0.0039 (19)	0.0100 (18)
Cu1	0.01451 (18)	0.01443 (18)	0.01021 (17)	−0.00137 (13)	0.00210 (13)	0.00019 (13)
F1	0.0661 (16)	0.0668 (16)	0.0353 (12)	0.0430 (13)	−0.0024 (11)	−0.0231 (11)
F2	0.0279 (13)	0.0601 (17)	0.146 (3)	0.0165 (12)	−0.0325 (15)	−0.0682 (18)
F3	0.0520 (14)	0.0250 (11)	0.0757 (17)	0.0113 (10)	0.0231 (12)	0.0097 (11)
F4	0.0368 (14)	0.0513 (15)	0.097 (2)	0.0078 (12)	−0.0289 (14)	−0.0069 (14)
F5	0.0514 (16)	0.0765 (19)	0.096 (2)	0.0380 (14)	0.0465 (15)	0.0410 (16)
F6	0.0356 (13)	0.0272 (12)	0.128 (3)	0.0030 (10)	0.0041 (14)	0.0199 (14)
N1	0.0175 (12)	0.0163 (12)	0.0154 (12)	0.0039 (10)	0.0006 (10)	−0.0015 (9)
N2	0.0166 (12)	0.0171 (12)	0.0158 (12)	0.0033 (10)	0.0008 (10)	0.0013 (10)
N3	0.0162 (12)	0.0186 (12)	0.0154 (12)	0.0036 (10)	0.0035 (10)	0.0036 (10)
N4	0.0368 (17)	0.0254 (15)	0.0197 (14)	−0.0056 (12)	−0.0015 (12)	0.0033 (11)
N5	0.0138 (12)	0.0168 (12)	0.0204 (13)	0.0022 (10)	0.0020 (10)	0.0003 (10)
N6	0.0182 (13)	0.0205 (13)	0.0149 (12)	0.0034 (10)	0.0041 (10)	0.0016 (10)
N7	0.0195 (13)	0.0208 (13)	0.0144 (12)	0.0051 (10)	0.0056 (10)	0.0033 (10)
N8	0.0257 (14)	0.0264 (14)	0.0241 (14)	0.0078 (12)	0.0076 (11)	0.0039 (11)
O1	0.0169 (11)	0.0192 (11)	0.0298 (12)	0.0019 (9)	0.0015 (9)	−0.0042 (9)
O2	0.0171 (12)	0.0361 (14)	0.0670 (18)	0.0071 (11)	−0.0103 (12)	−0.0199 (13)
O3	0.0530 (17)	0.0461 (16)	0.0358 (14)	0.0286 (13)	0.0241 (12)	0.0165 (12)
O4	0.0288 (12)	0.0361 (13)	0.0177 (11)	0.0163 (10)	0.0043 (9)	0.0006 (9)
O5	0.0500 (16)	0.0384 (14)	0.0158 (11)	0.0187 (12)	−0.0012 (10)	−0.0026 (10)
O6	0.0509 (16)	0.0250 (12)	0.0297 (13)	0.0135 (11)	−0.0041 (11)	0.0005 (10)
O7	0.0366 (14)	0.0455 (15)	0.0162 (11)	0.0165 (12)	0.0027 (10)	−0.0048 (10)
S1	0.0251 (4)	0.0207 (4)	0.0170 (4)	−0.0060 (3)	0.0001 (3)	0.0026 (3)
S2	0.0211 (4)	0.0172 (4)	0.0192 (4)	0.0004 (3)	0.0049 (3)	0.0025 (3)
S3	0.0135 (3)	0.0188 (4)	0.0229 (4)	0.0035 (3)	0.0007 (3)	−0.0032 (3)
S4	0.0304 (4)	0.0206 (4)	0.0154 (4)	0.0085 (3)	−0.0008 (3)	−0.0021 (3)

### Geometric parameters (Å, º)

**Table:**

C1—N2	1.317 (4)	C18—H18	0.9500
C1—C2	1.465 (4)	C19—N7	1.300 (4)
C1—S1	1.711 (3)	C19—C20	1.469 (4)
C2—N1	1.352 (4)	C19—S2	1.728 (3)
C2—C3	1.386 (4)	C20—N8	1.348 (4)
C3—C4	1.386 (4)	C20—C21	1.386 (4)
C3—H3	0.9500	C21—C22	1.380 (4)
C4—C5	1.374 (4)	C21—H21	0.9500
C4—H4	0.9500	C22—C23	1.379 (5)
C5—C6	1.393 (4)	C22—H22	0.9500
C5—H5	0.9500	C23—C24	1.384 (5)
C6—N1	1.335 (4)	C23—H23	0.9500
C6—H6	0.9500	C24—N8	1.328 (4)
C7—N3	1.296 (4)	C24—H24	0.9500
C7—C8	1.481 (4)	C25—F1	1.319 (4)
C7—S1	1.743 (3)	C25—F2	1.346 (4)
C8—N4	1.352 (4)	C25—F3	1.350 (4)
C8—C9	1.376 (4)	C25—S3	1.832 (3)
C9—C10	1.378 (4)	C26—F5	1.333 (5)
C9—H9	0.9500	C26—F4	1.349 (5)
C10—C11	1.377 (5)	C26—F6	1.367 (4)
C10—H10	0.9500	C26—S4	1.818 (4)
C11—C12	1.380 (5)	Cu1—N1	2.030 (2)
C11—H11	0.9500	Cu1—N2	2.032 (2)
C12—N4	1.352 (4)	Cu1—N5	2.040 (2)
C12—H12	0.9500	Cu1—N6	2.041 (2)
C13—N6	1.315 (4)	Cu1—O1	2.259 (2)
C13—C14	1.450 (4)	N2—N3	1.364 (3)
C13—S2	1.698 (3)	N6—N7	1.363 (3)
C14—N5	1.357 (4)	O1—H1W	0.7805
C14—C15	1.382 (4)	O1—H2W	0.8032
C15—C16	1.385 (4)	O2—S3	1.474 (2)
C15—H15	0.9500	O3—S3	1.452 (3)
C16—C17	1.384 (4)	O4—S3	1.429 (2)
C16—H16	0.9500	O5—S4	1.466 (2)
C17—C18	1.390 (4)	O6—S4	1.487 (2)
C17—H17	0.9500	O7—S4	1.424 (2)
C18—N5	1.334 (4)		
			
N2—C1—C2	119.3 (3)	C23—C22—H22	121.0
N2—C1—S1	113.2 (2)	C21—C22—H22	121.0
C2—C1—S1	127.4 (2)	C22—C23—C24	119.7 (3)
N1—C2—C3	123.8 (3)	C22—C23—H23	120.1
N1—C2—C1	112.3 (2)	C24—C23—H23	120.1
C3—C2—C1	123.9 (3)	N8—C24—C23	123.7 (3)
C2—C3—C4	118.5 (3)	N8—C24—H24	118.2
C2—C3—H3	120.8	C23—C24—H24	118.2
C4—C3—H3	120.8	F1—C25—F2	106.9 (3)
C5—C4—C3	118.4 (3)	F1—C25—F3	105.8 (3)
C5—C4—H4	120.8	F2—C25—F3	109.4 (3)
C3—C4—H4	120.8	F1—C25—S3	111.3 (3)
C4—C5—C6	119.7 (3)	F2—C25—S3	109.5 (2)
C4—C5—H5	120.1	F3—C25—S3	113.6 (2)
C6—C5—H5	120.1	F5—C26—F4	107.1 (3)
N1—C6—C5	122.9 (3)	F5—C26—F6	108.1 (3)
N1—C6—H6	118.6	F4—C26—F6	109.7 (3)
C5—C6—H6	118.6	F5—C26—S4	109.7 (3)
N3—C7—C8	124.4 (3)	F4—C26—S4	112.2 (3)
N3—C7—S1	114.4 (2)	F6—C26—S4	109.8 (2)
C8—C7—S1	121.2 (2)	N1—Cu1—N2	80.50 (10)
N4—C8—C9	124.2 (3)	N1—Cu1—N5	172.75 (10)
N4—C8—C7	113.7 (3)	N2—Cu1—N5	99.89 (10)
C9—C8—C7	122.1 (3)	N1—Cu1—N6	99.32 (10)
C8—C9—C10	118.9 (3)	N2—Cu1—N6	178.16 (10)
C8—C9—H9	120.6	N5—Cu1—N6	80.05 (10)
C10—C9—H9	120.6	N1—Cu1—O1	94.74 (9)
C11—C10—C9	118.6 (3)	N2—Cu1—O1	88.18 (9)
C11—C10—H10	120.7	N5—Cu1—O1	92.51 (9)
C9—C10—H10	120.7	N6—Cu1—O1	93.66 (9)
C10—C11—C12	119.0 (3)	C6—N1—C2	116.7 (2)
C10—C11—H11	120.5	C6—N1—Cu1	128.3 (2)
C12—C11—H11	120.5	C2—N1—Cu1	115.00 (18)
N4—C12—C11	123.9 (3)	C1—N2—N3	114.0 (2)
N4—C12—H12	118.0	C1—N2—Cu1	112.38 (19)
C11—C12—H12	118.0	N3—N2—Cu1	133.64 (18)
N6—C13—C14	118.8 (2)	C7—N3—N2	111.5 (2)
N6—C13—S2	114.3 (2)	C8—N4—C12	115.3 (3)
C14—C13—S2	126.9 (2)	C18—N5—C14	117.4 (2)
N5—C14—C15	123.6 (3)	C18—N5—Cu1	127.9 (2)
N5—C14—C13	112.7 (2)	C14—N5—Cu1	114.52 (19)
C15—C14—C13	123.6 (3)	C13—N6—N7	113.2 (2)
C14—C15—C16	118.2 (3)	C13—N6—Cu1	112.52 (19)
C14—C15—H15	120.9	N7—N6—Cu1	132.57 (19)
C16—C15—H15	120.9	C19—N7—N6	110.9 (2)
C17—C16—C15	118.8 (3)	C24—N8—C20	115.7 (3)
C17—C16—H16	120.6	Cu1—O1—H1W	116.9
C15—C16—H16	120.6	Cu1—O1—H2W	113.1
C16—C17—C18	119.6 (3)	H1W—O1—H2W	112.6
C16—C17—H17	120.2	C1—S1—C7	86.88 (14)
C18—C17—H17	120.2	C13—S2—C19	86.41 (14)
N5—C18—C17	122.3 (3)	O4—S3—O3	113.60 (14)
N5—C18—H18	118.8	O4—S3—O2	113.34 (15)
C17—C18—H18	118.8	O3—S3—O2	118.12 (17)
N7—C19—C20	124.1 (3)	O4—S3—C25	103.53 (15)
N7—C19—S2	115.2 (2)	O3—S3—C25	105.14 (17)
C20—C19—S2	120.7 (2)	O2—S3—C25	100.60 (15)
N8—C20—C21	124.8 (3)	O7—S4—O5	113.30 (14)
N8—C20—C19	113.1 (3)	O7—S4—O6	114.47 (15)
C21—C20—C19	122.1 (3)	O5—S4—O6	116.62 (14)
C22—C21—C20	118.0 (3)	O7—S4—C26	103.02 (17)
C22—C21—H21	121.0	O5—S4—C26	104.58 (18)
C20—C21—H21	121.0	O6—S4—C26	102.59 (16)
C23—C22—C21	118.1 (3)		
			
N2—C1—C2—N1	−0.2 (4)	C1—N2—N3—C7	0.4 (3)
S1—C1—C2—N1	175.6 (2)	Cu1—N2—N3—C7	−178.3 (2)
N2—C1—C2—C3	−177.1 (3)	C9—C8—N4—C12	−0.1 (5)
S1—C1—C2—C3	−1.2 (4)	C7—C8—N4—C12	179.3 (3)
N1—C2—C3—C4	−1.7 (5)	C11—C12—N4—C8	−0.6 (6)
C1—C2—C3—C4	174.8 (3)	C17—C18—N5—C14	−0.3 (4)
C2—C3—C4—C5	−0.3 (5)	C17—C18—N5—Cu1	174.3 (2)
C3—C4—C5—C6	1.7 (5)	C15—C14—N5—C18	2.4 (4)
C4—C5—C6—N1	−1.2 (5)	C13—C14—N5—C18	−177.2 (3)
N3—C7—C8—N4	177.9 (3)	C15—C14—N5—Cu1	−172.9 (2)
S1—C7—C8—N4	−1.8 (4)	C13—C14—N5—Cu1	7.5 (3)
N3—C7—C8—C9	−2.6 (5)	N2—Cu1—N5—C18	2.3 (3)
S1—C7—C8—C9	177.6 (2)	N6—Cu1—N5—C18	−175.8 (3)
N4—C8—C9—C10	0.7 (5)	O1—Cu1—N5—C18	90.9 (3)
C7—C8—C9—C10	−178.7 (3)	N2—Cu1—N5—C14	177.03 (19)
C8—C9—C10—C11	−0.6 (5)	N6—Cu1—N5—C14	−1.1 (2)
C9—C10—C11—C12	0.0 (5)	O1—Cu1—N5—C14	−94.4 (2)
C10—C11—C12—N4	0.6 (6)	C14—C13—N6—N7	179.7 (2)
N6—C13—C14—N5	−13.6 (4)	S2—C13—N6—N7	1.2 (3)
S2—C13—C14—N5	164.6 (2)	C14—C13—N6—Cu1	12.6 (3)
N6—C13—C14—C15	166.7 (3)	S2—C13—N6—Cu1	−165.88 (14)
S2—C13—C14—C15	−15.0 (4)	N1—Cu1—N6—C13	−178.8 (2)
N5—C14—C15—C16	−2.7 (5)	N5—Cu1—N6—C13	−6.2 (2)
C13—C14—C15—C16	176.9 (3)	O1—Cu1—N6—C13	85.7 (2)
C14—C15—C16—C17	0.7 (4)	N1—Cu1—N6—N7	17.3 (3)
C15—C16—C17—C18	1.3 (5)	N5—Cu1—N6—N7	−170.0 (3)
C16—C17—C18—N5	−1.5 (5)	O1—Cu1—N6—N7	−78.1 (3)
N7—C19—C20—N8	175.3 (3)	C20—C19—N7—N6	−179.8 (3)
S2—C19—C20—N8	−3.7 (4)	S2—C19—N7—N6	−0.7 (3)
N7—C19—C20—C21	−4.8 (5)	C13—N6—N7—C19	−0.4 (3)
S2—C19—C20—C21	176.1 (2)	Cu1—N6—N7—C19	163.4 (2)
N8—C20—C21—C22	−0.6 (5)	C23—C24—N8—C20	−1.2 (5)
C19—C20—C21—C22	179.5 (3)	C21—C20—N8—C24	1.1 (5)
C20—C21—C22—C23	0.2 (5)	C19—C20—N8—C24	−179.0 (3)
C21—C22—C23—C24	−0.3 (5)	N2—C1—S1—C7	0.4 (2)
C22—C23—C24—N8	0.9 (5)	C2—C1—S1—C7	−175.7 (3)
C5—C6—N1—C2	−0.8 (4)	N3—C7—S1—C1	−0.1 (2)
C5—C6—N1—Cu1	179.1 (2)	C8—C7—S1—C1	179.6 (3)
C3—C2—N1—C6	2.3 (4)	N6—C13—S2—C19	−1.3 (2)
C1—C2—N1—C6	−174.6 (3)	C14—C13—S2—C19	−179.6 (3)
C3—C2—N1—Cu1	−177.6 (2)	N7—C19—S2—C13	1.1 (2)
C1—C2—N1—Cu1	5.5 (3)	C20—C19—S2—C13	−179.8 (3)
N2—Cu1—N1—C6	173.7 (3)	F1—C25—S3—O4	170.5 (2)
N6—Cu1—N1—C6	−8.2 (3)	F2—C25—S3—O4	52.5 (3)
O1—Cu1—N1—C6	86.3 (3)	F3—C25—S3—O4	−70.1 (3)
N2—Cu1—N1—C2	−6.5 (2)	F1—C25—S3—O3	51.1 (3)
N6—Cu1—N1—C2	171.7 (2)	F2—C25—S3—O3	−66.9 (3)
O1—Cu1—N1—C2	−93.8 (2)	F3—C25—S3—O3	170.4 (2)
C2—C1—N2—N3	175.9 (2)	F1—C25—S3—O2	−72.1 (3)
S1—C1—N2—N3	−0.5 (3)	F2—C25—S3—O2	169.9 (3)
C2—C1—N2—Cu1	−5.1 (3)	F3—C25—S3—O2	47.2 (3)
S1—C1—N2—Cu1	178.51 (13)	F5—C26—S4—O7	179.2 (3)
N1—Cu1—N2—C1	6.1 (2)	F4—C26—S4—O7	60.3 (3)
N5—Cu1—N2—C1	−166.6 (2)	F6—C26—S4—O7	−62.1 (3)
O1—Cu1—N2—C1	101.2 (2)	F5—C26—S4—O5	−62.1 (3)
N1—Cu1—N2—N3	−175.2 (3)	F4—C26—S4—O5	178.9 (3)
N5—Cu1—N2—N3	12.2 (3)	F6—C26—S4—O5	56.5 (3)
O1—Cu1—N2—N3	−80.0 (3)	F5—C26—S4—O6	60.0 (3)
C8—C7—N3—N2	−179.9 (3)	F4—C26—S4—O6	−58.9 (3)
S1—C7—N3—N2	−0.1 (3)	F6—C26—S4—O6	178.7 (3)

### Hydrogen-bond geometry (Å, º)

**Table:**

*D*—H···*A*	*D*—H	H···*A*	*D*···*A*	*D*—H···*A*
O1—H1*W*···O6	0.78	1.95	2.721 (3)	169
O1—H2*W*···O2^i^	0.80	1.94	2.732 (3)	167
C6—H6···N7	0.95	2.33	3.146 (4)	143
C18—H18···N3	0.95	2.36	3.174 (4)	143

Symmetry code: (i) *x*−1, *y*, *z*.
